# Genetic diversity and origin of the feral horses in Theodore Roosevelt National Park

**DOI:** 10.1371/journal.pone.0200795

**Published:** 2018-08-01

**Authors:** Igor V. Ovchinnikov, Taryn Dahms, Billie Herauf, Blake McCann, Rytis Juras, Caitlin Castaneda, E. Gus Cothran

**Affiliations:** 1 Department of Biology, University of North Dakota, Grand Forks, North Dakota, United States of America; 2 Forensic Science Program, University of North Dakota, Grand Forks, North Dakota, United States of America; 3 Resource Management, Theodore Roosevelt National Park, Medora, North Dakota, United States of America; 4 Department of Veterinary Integrative Biosciences, College of Veterinary Medicine and Bioscience, Texas A&M University, College Station, Texas, United States of America; National Cheng Kung University, TAIWAN

## Abstract

Feral horses in Theodore Roosevelt National Park (TRNP) represent an iconic era of the North Dakota Badlands. Their uncertain history raises management questions regarding origins, genetic diversity, and long-term genetic viability. Hair samples with follicles were collected from 196 horses in the Park and used to sequence the control region of mitochondrial DNA (mtDNA) and to profile 12 autosomal short tandem repeat (STR) markers. Three mtDNA haplotypes found in the TRNP horses belonged to haplogroups L and B. The control region variation was low with haplotype diversity of 0.5271, nucleotide diversity of 0.0077 and mean pairwise difference of 2.93. We sequenced one mitochondrial genome from each haplotype determined by the control region. Two complete mtDNA sequences of haplogroup L were closely related to the mtDNA of American Paint horse. The TRNP haplotype B did not have close matches in GenBank. The phylogenetic test placed this sequence in a group consisting of two horses from China, one from Yakutia, and one from Italy raising a possibility of historical transportation of horses from Siberia and East Asia to North America. Autosomal STR loci were polymorphic and indicated that the TRNP horses were distinctly different from 48 major horse breeds. Heterozygosity, mean number of alleles, and other measures of diversity indicated that TRNP herd diversity was below that observed for most other feral herds and domestic breeds. Both mtDNA and STRs demonstrated that the existing genetic data sets of horses are insufficient to determine the exact origins of the TRNP horses. However, measures of nuclear and mitochondrial diversity have elucidated management needs. It is recommended that new genetic stock be introduced and that adaptive management principles are employed to ensure that unique mitochondrial lineages are preserved and genetic diversity is increased and maintained over time.

## Introduction

In the 1500s, 10,000 years after their extinction in the Americas, horses (*Equus caballus*) returned to the Western Hemisphere with Spanish conquistadors [1]. Subsequent exploration, colonization, trade, and free range livestock practices resulted in horses once again inhabiting many parts of the North American landscape. By the mid-1700s horses had been adopted into many Native American cultures and were widely used among tribes in the Great Plains from this point onward [2].

The westward advancement of Euro-American settlement of the continent during the 1800s, along with the advent of the cattle ranching industry, led to increasing numbers of horses of many origins and breeds on western rangelands, which in some cases became feral. These, along with tribal herds, are thought to be the source of many feral horse herds [3]. Today, some 77,000 feral horses roam the U.S. public lands in ten Western States [4], and multiple herds with varied introduction histories occur on government and private lands in other states, Canada, and Mexico [5].

The horse herd at Theodore Roosevelt National Park (TRNP) may have originated from multiple sources. The Native American horse trade centers such as the Missouri and Knife River villages of the Mandan, Hidatsa, and Arikara people of the Plains Village cultural tradition existed until at least 1850, and horse trade among other tribes was a common practice [6]. In 1883, a business man and entrepreneur based out of Medora, North Dakota purchased 250 horses confiscated from Lakota Sioux at Fort Buford. These horses entered the local ranching industry, and some were free-ranged in the badlands for breeding purposes [6]. From the mid-1880s onward the fate of these particular animals and their lineage is unclear. Free-ranging of ranch horses was a common practice at this time, and horses of many breeds were propagated among local ranches, with influxes of available stock from the railroad and from cattle drives along the Great Western Cattle Trail. Thus, it is unclear to what extent tribal ponies contributed to local feral herds over time, but it is likely that free-ranging animals comingled.

From the early 1900s until the establishment of the park in 1947 little is known about the status of feral horse herds in the area. The park began managing horses in the 1960s, and in 1966 it was thought that only 16 animals remained [6]. The herd was allowed to grow, with periodic roundups and auctions serving as the mechanism for maintaining horse numbers at a stocking rate appropriate for available forage. In the 1980s several stallions (including an Arabian, a Shire/Paint cross, a Quarter Horse, and three horses from the Bureau of Land Management, BLM) were introduced in an effort to improve genetics, and it is thought that some trespass horses may have entered the herd over time, despite the park boundary being fenced since 1956 [6]. Uncertain history and varied management practices over time have resulted in questions regarding origins, genetic diversity, and long-term genetic viability of the park herd. However, molecular tools may provide new understanding.

A number of studies have been accomplished to determine the genetic diversity and ancestry of feral horses in North America. In the first attempts to characterize feral horses, some populations were studied using blood groups and isoforms of enzymes. These techniques demonstrated that the feral horses of four eastern U.S. barrier islands (Shackleford Banks and Ocracoke Island in North Carolina, Cumberland Island in Georgia, and Assateague Island in Virginia—Maryland) and seven equine populations of Nevada and Oregon were not genetically unique in their allelic composition and did not significantly differ in heterozygosity from domestic breeds [7, 8, 9].

In the late 1990s autosomal short tandem repeats (STRs) replaced the protein marker systems and were able to detect changes in genetic variation caused by bottlenecks, genetic drift, isolation, non-random mating and inbreeding. A reduced number of STR alleles per locus and a significant heterozygous deficiency were found in the feral equine populations of Corolla and Ocracoke Islands off the North Carolina coast [10], on Sable Island of Nova Scotia, Canada [11, 12] and in the Granite Range Herd Management Area in Nevada [13]. The composition of STR alleles in North Carolina island horses was similar to New World Iberian horse breeds [10]. In contrast, the Sable Island horses represented a group of animals substantially diverged from any ancestral breeds according to genetic distance [11].

Mitochondrial DNA (mtDNA) has been analyzed to understand the origin and relationship of several feral equine herds in North America [14, 15, 16]. These studies were based on the analysis of a 5’ end section of the mtDNA control region [14]. The feral equine population on Sable Island represented by 21 animals demonstrated a very low genetic variation with four mtDNA haplotypes determined by three single nucleotide polymorphisms (SNPs). The very low haplotype diversity (0.27) was explained by the high level of inbreeding in that population [12, 14]. Other Canadian feral populations (actually no longer feral but living under domestication) indicated a higher mtDNA diversity with 11 haplotypes in 18 Newfoundland ponies and four haplotypes among 10 Lac La Croix horses corresponding to four mares that founded that herd in 1977 [14, 17]. Five other feral horse groups from the USA have been tested [16] with haplotype diversity ranging from 0.33 for the Sulphur herd in Utah to 0.93 for a group of Mustangs now in private hands. The Sulphurs are BLM managed horses where 2 haplotypes were observed out of 6 tested. In contrast the BLM herd from the Kiger herd area of Oregon had a haplotype diversity of 0.733 [16].

The first genetic studies of horses at TRNP were conducted during the 1990s, using blood groups and polymorphic red cell enzymes and other blood or serum proteins [18]. Analysis of the herd across four sampling periods, 1991, 1994, 1997, and 2000, indicated some losses of genetic variation over time at population and individual levels [18, 19]. In 2000, genetic variation also was evaluated at 12 STR loci for all sampling periods. This analysis demonstrated a significant decrease in heterozygosity across all the STRs in the TRNP herd since 1997 due to genetic drift and inbreeding [19], indicating the existence of possible problems with recessive mutation load and population fitness.

To date, mtDNA diversity and ancestry of the equine herd inhabiting TRNP have not been studied, and nuclear genetic analysis of the herd has not been updated since 2000. Consequently, current and complete genetic information has not been available to inform management strategies. To improve our understanding of the herd, we have identified three specific aims: 1) to examine mtDNA control region and nuclear genetic variation as an estimate of herd health and fitness 2) to determine the origin and relatedness of mtDNA lineages by comparison with a global reference of published sequences; and 3) evaluate relationships of park horses with domestic breeds and other feral herds through comparison to an autosomal STR genotype databases archived at Texas A&M University.

## Materials and methods

We collected mane and tail hair samples with follicles (n ≥ 30) from 196 horses during a roundup at TRNP in 2013. This sample represented a near census of the herd, since at that time a total of 214 horses existed in the park. We placed hair on collection cards (GeneSeek) and stored them at ambient temperature. Research was conducted under authorization of a National Park Service permit (TRNP-2013-SCI-0009), University of North Dakota (UND) Institutional Animal Care and Use Committee (Office of Laboratory Animal Welfare #A3917-01), and the UND Biosafety Committee (registration number IBC-201402-001). Data from herds managed by the Bureau of Land Management were used with permission from the BLM.

The herd has been studied by local enthusiasts for decades, and breeding information has been compiled by researchers conducting a concurrent contraception research project since 2009 [20]. Therefore, pedigrees based on field observations were available, and they indicated that horses were from 28 maternally related family groups with the number of available animals varying from 1 to 23 in 1 to 4 generations. Regardless, we attempted genetic analysis for all samples to avoid bias due to potential mistakes in the herd observational record.

### Mitochondrial DNA analysis

MtDNA was directly amplified from 1–3 follicles for each animal using the Terra PCR Direct Polymerase method (Clontech) according to the manufacturer’s protocol. To estimate the diversity of mtDNA lineages in the TRNP horse herd, we amplified a section of the mtDNA control region with the highest genetic variation encompassed by primers with 5’end coordinates corresponding to positions of 15,343 and 15,852 of the reference mtDNA sequence [21, 22]. This section included the area of the highest diversity in the horse mtDNA from position 15,500 to position 15,800 [23].

We utilized the mtDNA control region sequences to identify a subset of horses with different mtDNA haplotypes for amplification and sequencing of complete mitochondrial genomes. To obtain genomic sequence, we used 11 overlapping fragments with primers described by [23]. To improve the amplification of three overlapping segments of the horse mitochondrial genomes we designed and used three additional primer pairs (Table 1). The PCR products were evaluated using an agarose gel electrophoresis and were purified by DNA Clean & Concentrator-5 columns (Zymo Research) followed by quantification with a Nanodrop spectrophotometer and software V3.1.0 (Nanodrop Technologies). We then sequenced PCR fragments on an Applied Biosystems 3100 Genetic Analyzer using 33 nested oligonucleotides [23], carrying out reactions with the BigDye Terminator v.3.1 Cycle Sequencing kit (Thermo Fisher Scientific).

**Table 1:** PCR primers additionally designed to improve the amplification of three mtDNA sections.

Primer	Sequence (5' → 3')	5'end	mtDNA section
Ec-1650For	GTTCAAGCTCAACGACACAT	1650	2
Ec-3658Rev	GAGTGGTAGGAAGTTCTTTC	3658	
Ec-6509For	TAGGAGCAGTCTTCGCCATTATG	6509	5
Ec-8463Rev	CGGCGGTAATGTTAGCGGTTAG	8463	
Ec-15330For	CCCTGTAGTATATCGCACAT	15330	11
Ec-573Rev	AGTAGTACTCTGGCGAATAG	573	

The visual inspection of the chromatograms of DNA sequences was conducted using BioEdit 5.0.6 [24]. The sequences were aligned with the horse reference mtDNA (GenBank accession number X79547) using MEGA7 [21, 25]. All the DNA polymorphisms were independently verified by two researchers. We determined genetic diversity indexes in 29 horses representing all maternally related groups living in TRNP with one mtDNA control sequence from every family group using ARLEQUIN 3.5.2.2 [26].

We conducted primary identification of mtDNA lineages using a BLAST search at https://blast.ncbi.nlm.nih.gov/Blast.cgi of the matched sequences and comparison of their haplogroup affiliation according to nomenclature proposed by [23]. Haplogroups of the complete mtDNA sequences were confirmed by the phylogenetic analysis using Bayesian, neighbor-joining, and maximum parsimony methods. We employed MrBayes 3.2.6 [27] for estimation of Bayesian phylogeny using the HKY85 (Hasegawa-Kashino-Yano-1985) model and the parameters described by [23] and MEGA 7 to support the positions of the TRNP horse mtDNA sequences in the Bayesian phylogeny by the neighbor-joining and maximum parsimony phylogenetic analyses [25]. The phylogenetic analyses demonstrated the position of the complete mitochondrial genome sequences found in the TRNP horses in the global horse mtDNA phylogeny comprising the three mtDNA sequences of the Park’s horses, the reference horse mtDNA [20], 83 mtDNA sequences belonging to all main haplogroups from [23], and the mtDNA of a donkey *Equus asinus* (GenBank accession number NC_001788) [28] used as a root.

Program mcmctree in PAML 4.9 [29] was used to estimate divergence time in a set of modern horse mtDNAs sampled from haplogroups B and L [23] so that internal nodes had a 1.0 posterior probability on the Bayesian phylogenetic tree. The molecular clock remained linear over time according to the previous studies [23, 30, 31]. We assumed divergence time between donkey (*Equus asinus*) and horse mtDNAs of 2 million years with the range of 3.0–1.0 million years [23, 30], the split time for the mtDNAs of the horse breeds of 150,000–40,000 [23, 30] and used the HKY85 model with alpha equals 0.2 for gamma rates at sites.

### Autosomal STR analysis

To get autosomal STR profiles, total DNA from hair follicle samples was extracted using the PUREGENE^®^ DNA purification kit following the manufacturer’s protocol (Qiagen). We tested 12 autosomal STR markers (AHT4, AHT5, ASB2, ASB17, ASB23, HMS6, HMS7, HMS3, HTG4, VHL20, HTG10, and LEX33) distributed on 10 chromosomes of the horse genome. Information about the loci location, primer sequences and allele sizes can be found in [32]. STR genotyping was performed following the method described by [33].

Various measures of genetic variation were calculated. The measures are observed heterozygosity (Ho) which is the proportion of loci shown to be heterozygous in individuals, Hardy-Weinberg expected heterozygosity (He) which is based upon allele frequencies, inbreeding coefficient (Fis) which is calculated as 1-(Ho/He), effective number of alleles (Ae) which is a measure of allelic diversity, total number of variants (TNV) which is the number of different alleles found in the population, mean number of alleles (MNA) per locus, number of rare alleles (Ra) which are alleles with a frequency of 0.05 or less in the population, and the percentage of rare alleles (%Ra) compared to total alleles. The rare alleles measures are indicators of variation at risk of loss.

We determined genetic relationship of TRNP horses to a variety of horse breeds using the Majority-rule consensus of a Restricted Maximum Likelihood (RML) tree. The breeds used in the analysis had been tested in previous studies and represented most major horse breed groups. We calculated chord distances from 100 bootstraped frequency datasets using PHYLIP 3.69 [34] and then visualized trees using MEGA6 [35], with Przewalski horse (n = 31) as the outgroup.

We compiled pictorial information about the genetic relationship among breeds using Principal Coordinates Analysis (PCoA) as implemented by GENETIX 4.05 [36]. For additional comparison, STRUCTURE 2.3.3 software [37] was used to study the patterns among the TRNP herd and nine other groups which contained very distinct populations, globally, with burn-in values of 10,000 and 50,000 MCMC repetitions. Each run for the K value (K = 2 to K = 11) were repeated 10 times. No set out-group was used as each population was anticipated to be drastically different. We then used the software CLUMPP [38] and DISTRUCT [39] to align multiple replicates and display the results, respectively. Finally, we determined the best number of clusters based upon ΔK value [40] using the Structure Harvester application [41].

## Results

We successfully amplified and sequenced mtDNA control region segments for 187 horses, for which only three variant haplotypes were observed (Table 2). Two of these lineages belong to haplogroup L with SNPs at positions 15,494, 15,496, 15,534, 15,603, and 15,649 specific for that haplogroup [23]. Both mtDNA sequences differed in one SNP at position 15,827 with a guanine in the lineage L1 and an adenine in the lineage L2 as also observed in the horse reference sequence [21]. The third lineage was diagnosed as belonging to haplogroup B based on the BLAST search (see below) because haplogroup B has no characteristic SNPs in the 15,403–15,830 section of the mtDNA (Table 2).

**Table 2:** Genetic variation of the 5’ portion of the mtDNA control region in horses living in Theodore Roosevelt National Park. The sequences were aligned with the horse reference mtDNA sequence (GenBank accession number X79547). Numbers show transitions.

Lineage	Single nucleotide polymorphisms
B1	15495, 15650, 15666, 15720, 15826
L1	15494, 15495, 15496, 15534, 15602, 15603, 15649, 15720, 15771, 15827
L2	15494, 15495, 15496, 15534, 15602, 15603, 15649, 15720, 15771

Lineage L1 was found in 19 maternally related groups, representing 123 horses. Lineage L2 was carried by 33 animals in 6 family groups, and haplogroup B was detected in 31 horses in 4 family groups. Although the L1 control region is the most common sequence among the TRNP horses, our BLAST search revealed the reverse picture among the global database of horse mtDNA sequences deposited to GenBank. The L1 control region sequences from TRNP only matched to two sequences in GenBank belonging to an American Paint horse (JN398421) [23] and an unspecified East Asian horse (JQ340100). The BLAST search returned 31 identical sequences for the L2 control region sequence found in domestic horses from Britain, Ireland, Poland, southeast Europe, Central Asia, Iran, China and Japan and in Lippizan, Thoroughbred, Percheron, Appaloosa, Arabian, Akhal-Teke, Lusitano, Irish Draught, Baise, Paint Horse, Fell and Welsh pony breeds, as well as other unspecified breeds. The B control region sequence also had a global distribution, with 22 hits in GenBank representing domestic horses in Ireland, central Europe, south and southeast Europe, Russia (including 5 mtDNA sequences from Yakutia in central Siberia), China, and Japan, as well as Irish Draught, Thoroughbred, Lusitano, Arabian, Vyatskaya, Westphalian, Russian Riding, Mongolian Native and unspecified breeds.

Using the ARLEQUIN program, the population diversity indexes were determined in 29 horses representing all maternally related groups living in TRNP with one mtDNA control sequence from every family group. We determined that the haplotype diversity was 0.5271 ± 0.0864, nucleotide diversity was 0.0077 ± 0.0046, and mean pairwise difference was 2.93 ± 1.58 among mtDNA from maternally related groups (Table 3).

**Table 3:** The mtDNA diversity statistics in the feral horse populations.

	TRNP	Sable Island	Lac La Croix	Newfoundland	Assateague Island
Haplotype diversity	0.5271 ± 0.0864	0.27 ± 0.12	0.64 ± 0.15	0.96 ± 0.08	
Mean p/w differences	2.93 ± 1.58	0.286 ± 0.317	5.867 ± 3.062	4.392 ± 2.275	
Nucleotide diversity	0.0077 ± 0.0046	0.0008 ± 0.0004	0.0155 ± 0.0033	0.0148 ± 0.0028	0.011 ± 0.006

The mtDNA control region data was unable to determine the origin and relationship of the TRNP feral horses due to a low number of phylogenetically informative sites within the short sequences. This pitfall prevents a high statistical support within the global mtDNA phylogenetic tree of domestic horses with most of the nodes remaining unresolved. To solve that issue, the entire mitochondrial genomes should be considered [23].

Three horses with the numbers of 201346, 201016, and 201230 carrying mtDNA haplotypes L1, L2, and B, respectively were selected for sequencing of mitochondrial genomes. These three complete mtDNA sequences varied in 86 polymorphic positions with 62 SNPs in the coding portion and 24 in the control region (S1 Table). All three mitochondrial genomes carried diagnostic mutation motifs corresponding to haplogroups L and B [23]. Only two of these SNPs, in positions 15,827 and 16,145, within the control region distinguish the L1 and L2 lineages, confirming their close relationship. Position 16,145 is located within a short tandem repeat region between the 16,129 and 16,360 nucleotide positions, which is excluded from many phylogenetic reconstructions of horse mtDNA due to a great variability of this repeat sequence. All 84 other polymorphic SNPs differentiate lineages L and B. This number of SNPs correlates with an average amount of 82.0 ± 30.7 nucleotide differences between two randomly selected sequences belonging to the major haplogroups [23].

Most variation between the B and L mtDNA lineages in the TRNP horses was found in the ND5 and ND4 genes with 10 and 9 SNPs, respectively. It was previously reported that these genes are among most polymorphic in the horse mtDNA [42]. Three polymorphic deletions of single nucleotides were found in tRNA genes including 1,386delT at tRNA-Val gene and 5,240delA and 5,279delA at tRNA-Cys in haplotypes L1 and L2. These deletions were previously reported in other horses as well [23].

BLAST searches indicated that the three mitochondrial genomes found in the TRNP horses are unique, with no identical sequences reported in the GenBank database (S2 Table). The mtDNA sequence of the American Paint horse with the accession number JN398421 [23] has one deleted thymine at position 15,388 distinguishing it from 201346 (haplotype L1). The American Paint horse mtDNA also differs in 15,388 del T, 15,827, and 16,145 from 201016 (haplotype L2). Other sequences similar to the mtDNA of horses 201346 and 201016 found in GenBank were a Thoroughbred horse from China (KC203002) and an unspecified Italian breed (JN398425) [23].

In contrast, the mitochondrial genome of horse 201230 belonging to haplogroup B does not have close matches in GenBank as do the L lineages. The closest mtDNA sequence is carried by a Thoroughbred racing horse from China (KC202958) and differs from 201230 mtDNA in 6 SNPs at positions 5,016, 5,098, 10,125, 10,126, 10,737, and 16,391 and four single nucleotide deletions in sites 5,240, 5,279, 15,388, and 16,407. Other closely related sequences to the horse 201230 mtDNA were obtained from the unspecified Italian breed horse (JN398390) [23], the Yunnan horse (JQ340126) from China, and the Yakutia horse (KT368729) from central Siberia, Russia [31].

**Fig 1:**
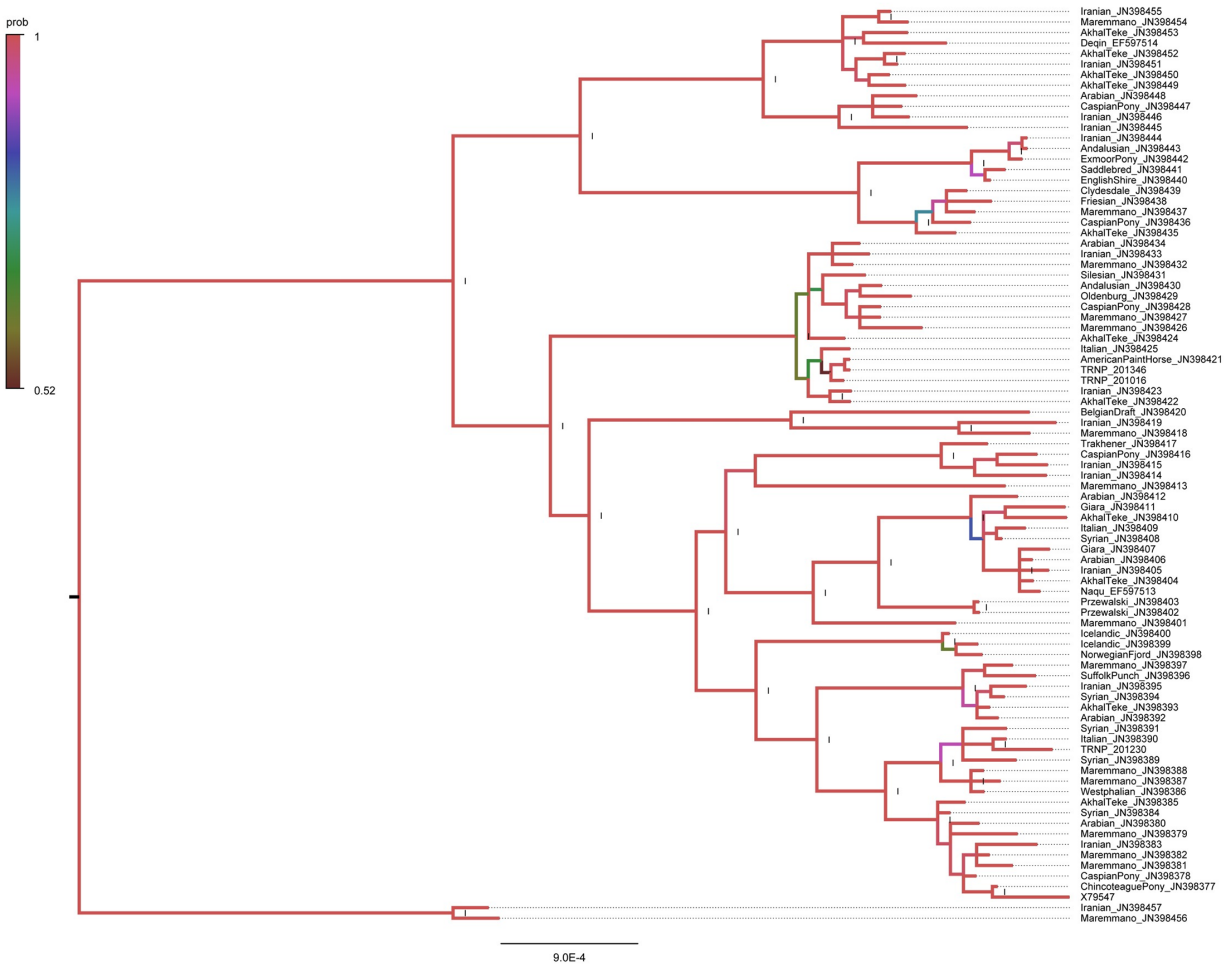
Bayesian inference phylogenetic tree demonstrating the phylogenetic relationship of mitochondrial genomes of three mtDNA lineages found in the TRNP horses, and the mtDNAs of 84 modern horses. The donkey mitochondrial genome sequence (NC_001788) used as an outgroup is not displayed. Numbers shown by the Roman numeral I indicates the clades with the highest posterior probability value. The color scale refers to the clades with the posterior probabilities between 0.5 and 1.

Phylogenetic analyses indicated strong statistical support for placing the horse 201230 mtDNA in haplogroup B and the mtDNA sequences of horses 201346 and 201016 within haplogroup L (Fig 1). The similar position for three mtDNA sequences of the TRNP horses was also demonstrated by neighbor-joining and maximum parsimony methods.

Divergence time of haplogroups B and L was estimated to be 90,000 years with a 95% confidence interval of 30,000–150,000 years that well corresponds to the Bayesian age estimate calculated using all substitutions in the 83 horses belonging to all main haplogroups [22]. The age of haplogroup B is 30,000 years with a 95% confidence interval of 10,000–70,000 years and the age of haplogroup L is 20,000 years with a 95% confidence interval of 0.0–50,000 years. We estimated that the split between the horse 201230 mtDNA and JN398390, the closest sequence within haplogroup B in Fig 1 occurred 10,000 years ago with a 95% confidence interval of 0.0–30,000 years. In haplogroup L the separation between the horse 201016 mtDNA from the group consisting of the horse 201346 mtDNA and JN398421 happened 10,000 years ago with a 95% confidence interval of 0.0–20,000 years. Finally, the split between the horse 201346 mtDNA and JN398421 is a result of the very recent divergence (0.0 with a 95% confidence interval of 0.0–10,000 years).

All autosomal STR loci tested were polymorphic and did not deviate from HWE in the breeds or the TRNP herd tested (P<0.05). Observed heterozygosity (Ho) and mean number of alleles (Ae) were lower for the TRNP herd than the mean values for BLM herds (Table 4) and most domestic breeds except Cleveland Bay and Friesian breeds (Table 4). While the situation with inbreeding coefficient (Fis) was not so obvious, Fis values for horses tend to not be statistically significantly different from zero. Values of other diversity measures (such as He, Ae, TNV, MNA, Ra, %Ra) shown in Table 2 for the TRNP herds and selected other feral herds and domestic horse breeds. For all measures except those related to rare alleles, the TRNP herd had values well below both the Feral horse mean and the Domestic breed means.

**Table 4:** Comparison of genetic diversity between the Theodore Roosevelt National Park horse herd, other feral herds, and selected domestic breeds.

	*N*	*Ho*	*He*	*Fis*	*Ae*	*TNV*	*MNA*	*Ra*	*%Ra*
**Feral Herds**									
**Theodore Roosevelt NP**	**197**	**0.621**	**0.624**	**0.005**	**3.040**	**60**	**5.000**	**15**	**0.250**
Pryor Mountains MT	103	0.757	0.762	0.007	4.507	79	6.583	15	0.190
Fox Hog CA	115	0.717	0.730	0.018	4.031	89	7.417	33	0.371
Kiger Herd OR	56	0.722	0.707	-0.022	3.710	80	6.667	24	0.300
McCullough Peaks WY	50	0.755	0.715	-0.055	3.823	76	6.333	24	0.316
Stone Cabin NV	50	0.763	0.777	0.015	4.712	92	7.667	31	0.337
**Domestic Breeds**									
Cleveland Bay	47	0.610	0.627	0.027	2.93	59	4.92	16	0.271
American Saddlebred	576	0.740	0.745	0.007	4.25	102	8.50	42	0.412
Andalusian	52	0.722	0.753	0.041	4.26	79	6.58	21	0.266
Arabian	47	0.660	0.727	0.092	3.81	86	7.17	30	0.349
Exmoor Pony	98	0.535	0.627	0.146	2.87	66	5.50	21	0.318
Friesian	304	0.545	0.539	-0.011	2.56	70	5.83	28	0.400
Irish Draught	135	0.802	0.799	-0.003	5.19	102	8.50	28	0.275
Morgan Horse	64	0.715	0.746	0.041	4.19	92	7.67	33	0.359
Suffolk Punch	57	0.683	0.711	0.038	3.88	71	5.92	13	0.183
Tennessee Walker	60	0.666	0.693	0.038	3.66	87	7.25	34	0.391
Thoroughbred	1195	0.734	0.726	-0.011	3.92	69	5.75	18	0.261
**Feral Horse Mean**	**126**	**0.716**	**0.710**	**-0.012**	**3.87**	**72.68**	**6.06**	**17**	**0.222**
Standard Deviation		0.056	0.059	0.071	0.66	13.02	1.09	7.98	0.088
Minimum		0.496	0.489	-0.284	2.15	37	3.08	0	0
Maximum		0.815	0.798	0.133	5.25	96	8.00	33	0.400
**Domestic Horse Mean**	**80**	**0.710**	**0.720**	**0.012**	**4.01**	**80.88**	**6.74**	**23.8**	**0.283**
Standard Deviation		0.078	0.071	0.086	0.74	16.79	1.40	10.1	0.082
Minimum		0.347	0.394	-0.312	1.78	26	2.17	0	0
Maximum		0.822	0.799	0.211	5.30	119	9.92	55	0.462

N, sample size; Ho, observed heterozygosity; He, expected (H-W) heterozygosity; Fis, inbreeding coefficient; Ae, effective number of alleles; TNV, total number of variants observed; MNA, mean number of alleles; Ra, number of rare alleles (frequency equal or below 0.05); %Ra, percentage of rare alleles.

**Fig 2:**
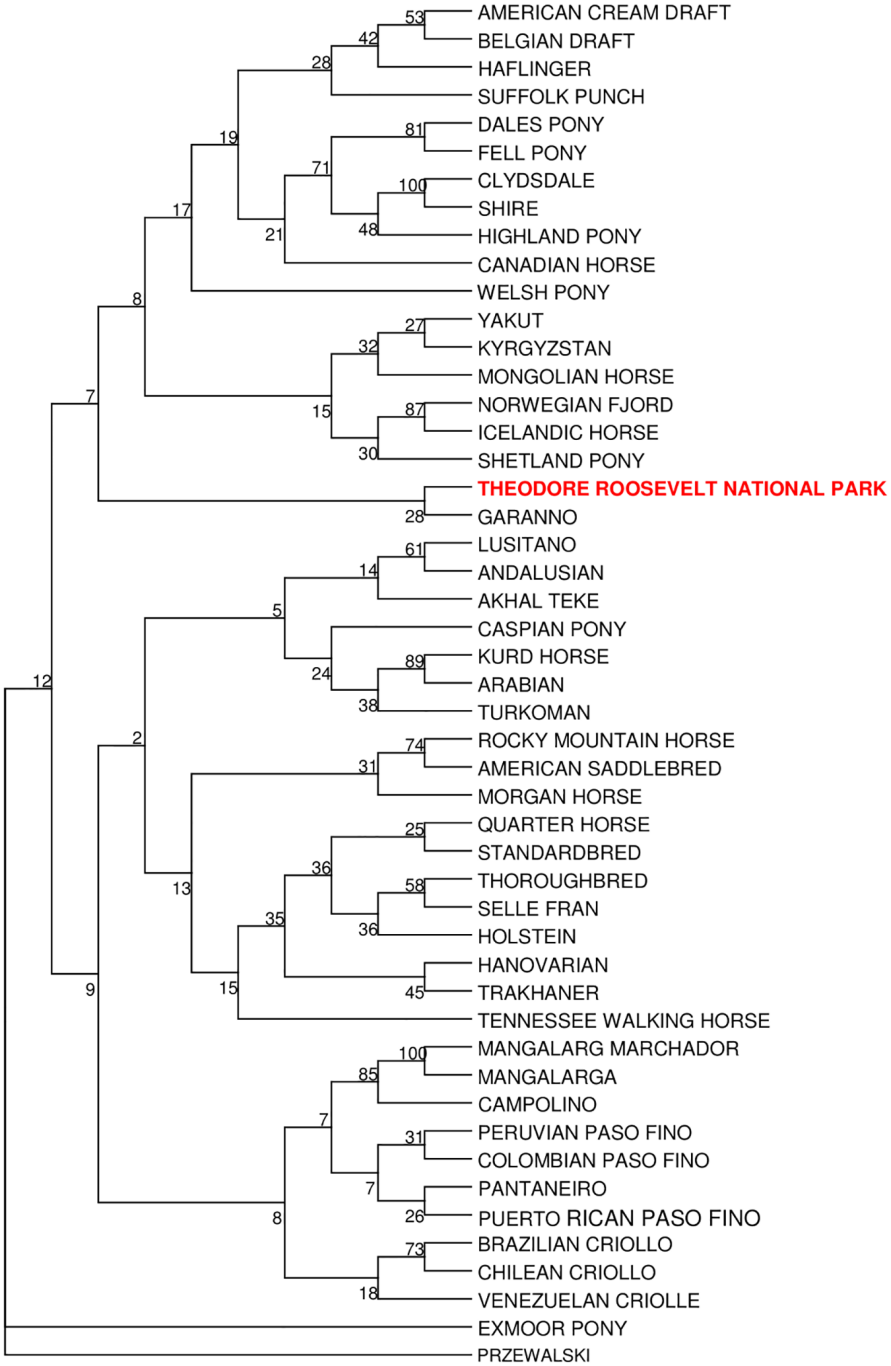
Restricted Maximum Likelihood tree of nuclear genetic relationships Theodore Roosevelt National Park horses in comparison to 46 domestic breeds and Przewalski Horse (used as an outgroup). Numbers at split represent probability based on bootstrapping.

**Fig 3:**
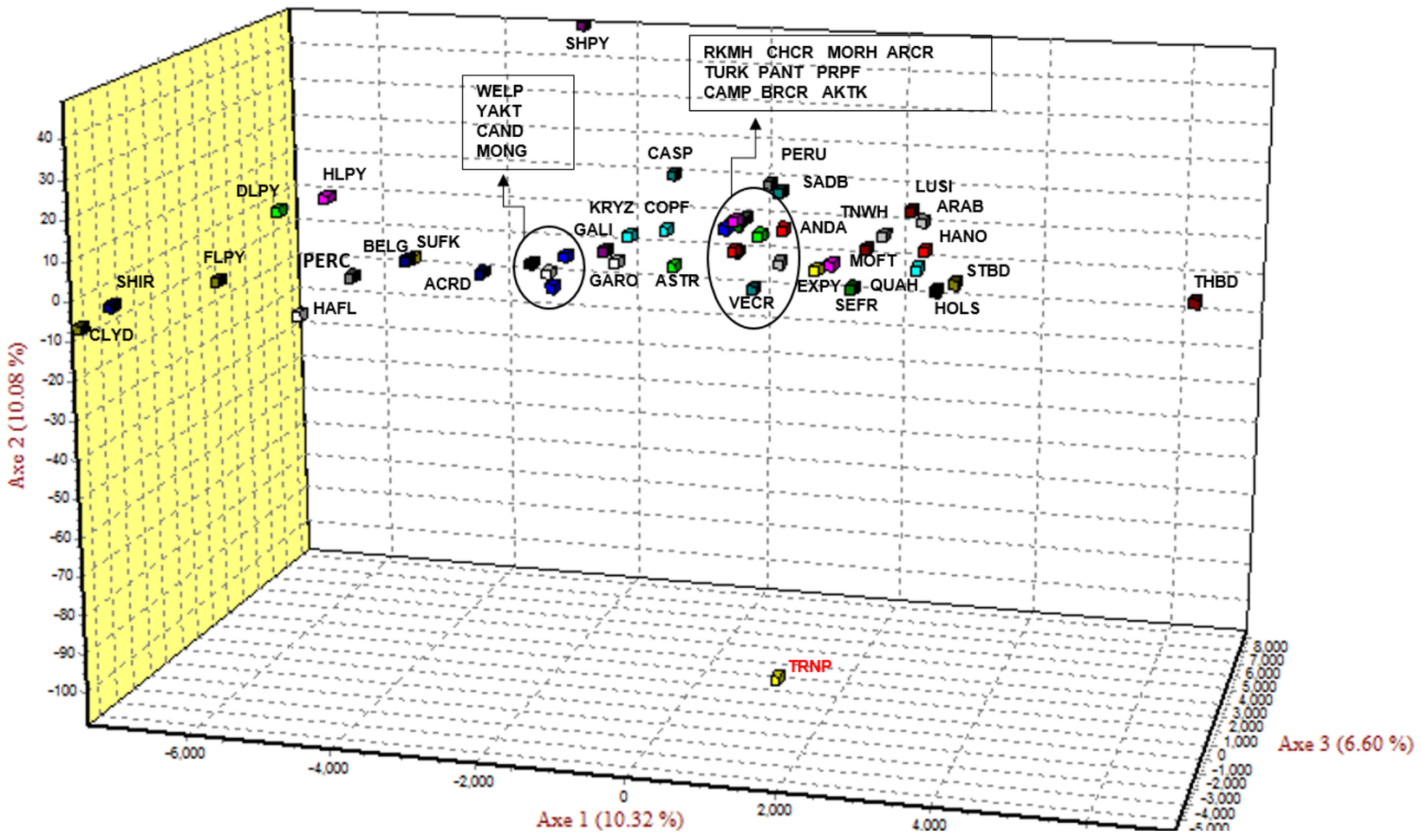
Principal Coordinates Plot of nuclear genetic relationships for horses at Theodore Roosevelt National Park in comparison to select domestic breeds. ACRD, American Cream Draft; AKTK, Akhal Teke; ANDA, Andalusian; ARAB, Arabian; ASTR, Asturcon; BELG, Belgian Draft; BRCR, Brazillian Criollo; CAMP, Campolino; CAND, Canadian; CASP, Caspian; CHCR, Chilean Criollo; CLYD, Clydsedale; COPF, Colombian Paso Fino; DLPY, Dales Pony; FLPY, Fell Pony; GALI, Galiceno; GARO, Garrano; HAFL, Haflinger; HANO, Hanoverian; HLPY, Highland Pony; HOLS, Holstein; KRYZ, Kyrgyztan; LUSI, Lusitano; MOFT, Missouri Fox Trotter; MONG, Mongolian Horse; MORH, Morgan Horse; PANT, Pantaniero; PERC, Percheron; PERU, Peruvian Paso; PRPF, Puerto Rican Paso Fino; QUAH, Quarter Horse; RKMH, Rocky Mountain Horse; SADB, American Saddlebred; SEFR, Selle Francais; SHIR, Shire; SHPY, Shetland Pony; STBD, Standardbred; SUFK, Suffolk Punch; THBD, Thoroughbred; TNWH, Tennessee Walking Horse; TRNP, Theodore Roosevelt National Park; TURK, Turkoman; VECR, Venezuelian Criollo; WELP, Welsh Pony; YAKT, Yakut.

The RML tree paired the Theodore Roosevelt herd with the Garanno, a semi-feral horse breed from Portugal (Fig 2). This branch was between two large clades that together essentially represent all the domestic breeds analyzed with the exception of the Exmoor Pony which falls between the outgroup and all other breeds. The PCoA plot of Theodore Roosevelt samples and 48 diverse horse breeds indicated that the Theodore Roosevelt samples were distinctly different. Just over 10% of the genetic variance was explained by the first two axes (Fig 3). The Theodore Roosevelt samples followed similar first-axis patterns as the Iberian derived breeds but were widely different on the second axis. Thus, although the feral TRNP horses showed some association with at least one Iberian breed in the RML tree, as shown by the PCoA the relationship was not close.

**Fig 4:**
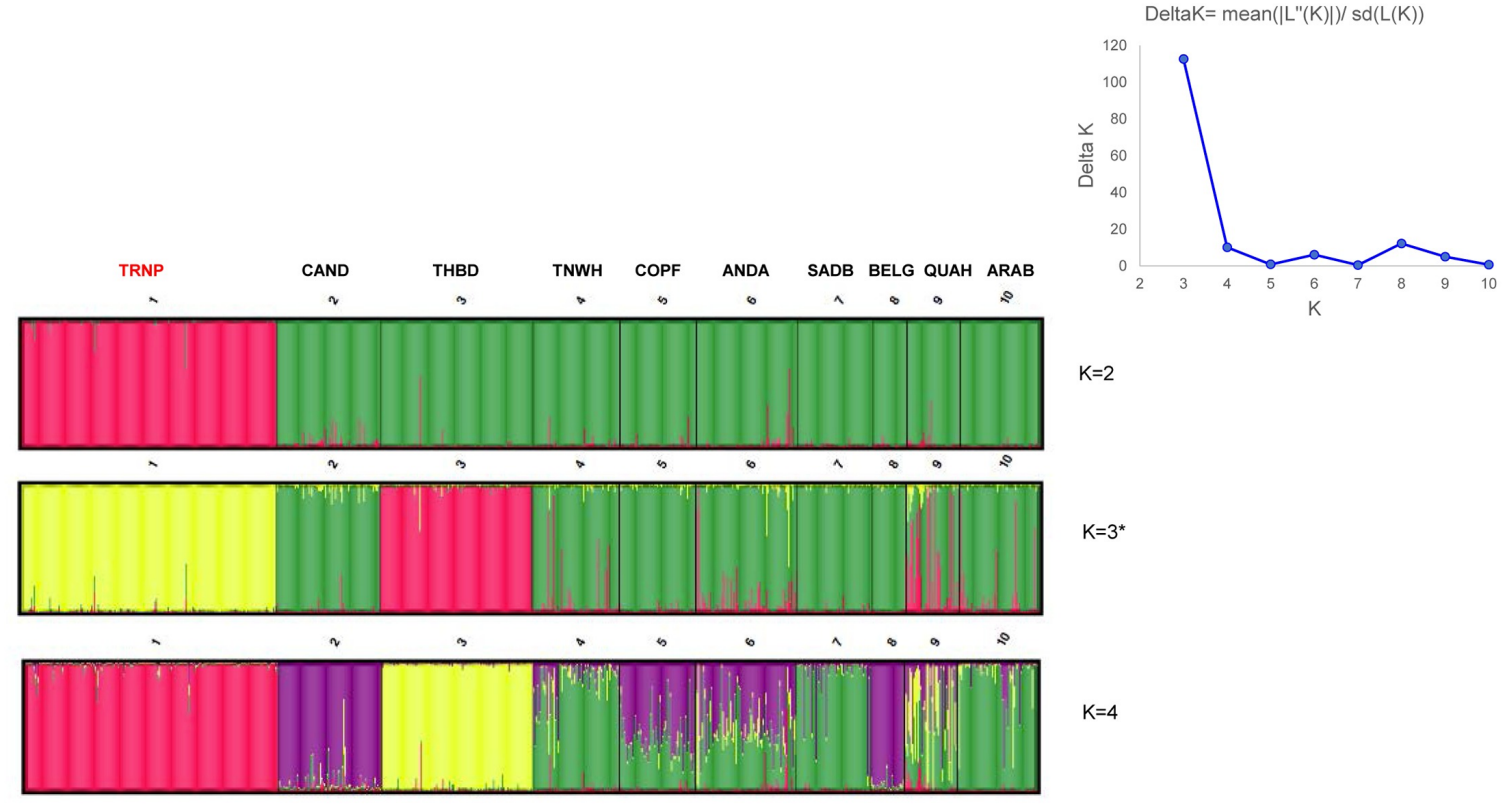
Barplot from STRUCTURE analysis of Theodore Roosevelt National Park horses and nine domestic breeds found in North America according to the following abbreviations: CAND, Canadian Horse; THBD, Thoroughbred; TNWH, Tennessee Walker; COPF, Colombian Paso Fino; ANDA, Andalusian; SADB, American Saddlebred; BELG, Belgian Draft; QUAH, Quarter Horse; ARAB, Arabian. Note: K = 3 (denoted with asterisk) was deemed the best result based on ΔK evaluation (insert).

**Fig 5:**
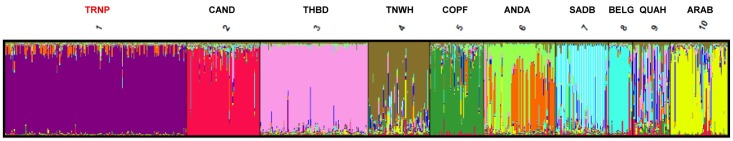
Barplot of K = 11 STRUCTURE result for Theodore Roosevelt National Park horses and nine domestic breeds according to the following abbreviations: CAND, THBD, TNWH, COPF, ANDA, SADB, BELG, QUAH, and ARAB (Abbreviations same as Fig 4). Note: presented to demonstrate that at the highest possible resolution, park horses still form their own genetic cluster.

The STRUCTURE analysis of the Theodore Roosevelt samples with 9 other breeds (Canadian, Thoroughbred, Tennessee Walker, Columbian Paso Fino, Andalusian, American Saddlebred, Belgian Draft, Quarter Horse, and Arabian) with no outgroup using K = 11 initially showed that ΔK was at K = 3 where three clusters were formed (Fig 4). The main group (green) was comprised of every breed except the Thoroughbred and Theodore Roosevelt samples. The Theodore Roosevelt group (yellow in K = 3) showed early separation during STRUCTURE analysis. Analysis was carried out to K = 11 (Fig 5) which once more indicated the Theodore Roosevelt samples had no integration of the other breeds in their cluster.

## Discussion

Numerous herds of feral horses roam government, public and private lands in the U.S. However, understanding of their origins and genetic variation is fragmentary. The equine herds living in the U.S. national parks represent a model that can be used to study the historic value as well as the genetic diversity and health of these animals and to establish the conservation strategy for the future.

We used two traditional types of genetic markers, autosomal STRs and mtDNA control region, to evaluate the TRNP herd’s genetic variation and origins. To date, the most extensive published study of a feral equine population in North America was done in Canada for Sable Island horses [11]. The only study of the U.S. feral horse population involving both types of genetic markers was carried out in the Maryland herd of Assateague Island National Seashore [43]. The comparison of population statistics with other feral herds in North America helped to trace demographic reasons behind higher or lower genetic diversity.

The diversity of the mtDNA control region of the TRNP herd (Table 3) was higher than in the Sable Island horses having the four close haplotypes distinguished by three variable sites [14]. The lowest mtDNA diversity in Sable Island among the Canadian feral horse populations [14] was caused by the high amount of inbreeding, the small population size and the isolation from introgression for at least 11–12 generations [11].

In contrast, three other feral equine populations with three mtDNA control region haplotypes in the Maryland herd, 11 haplotypes in the Newfoundland ponies and four haplotypes in the Lac La Croix had higher mtDNA diversity than the TRNP horses [14, 43]. However, the Newfoundland Pony and the Lac La Croix horses are not truly feral any more. The five groups tested in [16] also all had higher diversity than the TNRP herd (Florida Cracker horse had 2 haplotypes from 3 samples, Kiger herd 3 haplotypes from 6 samples, Spanish Mustang Registry horses 3 haplotypes from 4 samples, Sulphur herd 2 haplotypes from 6 samples and Mustang group 5 haplotypes from 6 samples). The higher diversity in these populations can be explained by several processes that influence population genetic variation and that may be considered for management decisions in Theodore Roosevelt National Park. For instance, the Lac La Croix population’s diversity was shaped by the recent admixture of mustangs [12]. The revival of the Newfoundland population after a severe bottleneck in the 1980s was caused by crossing with Standardbreds and Clydesdales and through gene flow between the Newfoundland and Saint-Pierre et Miquelon feral populations [12, 14]. In the Maryland herd of Assateague Island the genetic diversity was affected by interplay between isolation and a higher effective population size in a small population [43]. The Spanish Mustang Registry horses and the Mustang group are very outcrossed and the Sulphur herd has a fairly large population size. However, the Florida Cracker horse and the Kiger herd have relatively small population sizes although the Kiger herd has high STR variation.

The observed reduced genetic diversity in the TRNP horses was due to the population bottleneck in the 1960s and a small number of introduced animals after that. Among TRNP horses we found only three haplotypes among 187 animals based on the mtDNA control region sequencing. The three mtDNA control sequences were unable to provide sufficient phylogenetic information and posit them on the global horse mtDNA tree with confidence. BLAST search demonstrated a global distribution of similar mtDNA control sequences especially of L2 and B haplotypes across Eurasia and different breeds.

In contrast to the control region, the whole mitochondrial genome sequencing demonstrated that the TRNP horses possessed three unique complete mtDNA lineages not reported in GenBank to date. The phylogenetic analysis indicated that the three mtDNA sequences found in the TRNP horses do not have monophyletic origins. The presence of divergent mtDNA haplotypes L and B clearly confirmed that the current horse herd at TRNP originated from at least two sources.

The substantial part of the TRNP herd carried two mtDNA lineages named for this study as L1 and L2 that belonged to haplogroup L [23] and were very similar to each other. Two lineages were found in 25 of 29 (86.2%) maternally related groups combining 156 of 187 (83.4%) animals. Nowadays, in the Old World haplogroup L is the most frequent in Europe (38.1%) followed by the Middle East (22.4%) and the rest of Asia (13.5%) [23]. However, the highest frequency of this haplogroup was reported in North America (58.7%) and in South America (47.9%) [23], probably due to the founder effect when this lineage was introduced to the Americas by the first European people followed by an export of a large number of animals to the continent’s countries.

Four family groups (13.8%) with 31 animals (16.6%) bore the mtDNA haplogroup B with distinct differences from lineages L1 and L2. The closest mtDNA sequence was found in a Thoroughbred racing horse from China (KC202958) but that sequence was distinct from the TRNP B lineage in 6 SNPs and four single nucleotide deletions. The entire BLAST cluster also included complete mtDNA sequences of the Italian horse of an unspecific breed (JN398390) [23], the Yunnan horse (JQ340126) from China, and the Yakutia horse (KT368729) from central Siberia, Russia [31]. Based on the control region comparison, haplogroup B seems to be most frequent in North America (23.1%), with lower frequencies in South America (12.68%) and the Middle East (10.94%) and Europe (9.38%) [23]. Although the frequency of this lineage is low (1.7%) in the Asian sample of 587 horses [23], this lineage was found in the Bronze Age horses from China and South Siberia [44]. The unexpected link between the TRNP lineage B and the horses from Asia may be explained by insufficient sampling of horse mitochondrial genome variation in Europe and North America. However, this observation leads to an exciting hypothesis about a link between Siberian—East Asian and part of North American horses and a new route of exporting horses to North America. The intriguing possibility of gene flow from Siberian horses of Yakutia to Canadian horses was suggested from the genetic variation observed in the wild horse population living in the remote Brittany Triangle region of British Columbia [45].

To prove this link, extensive sampling of DNA for complete mtDNA sequencing and microsatellite profiling should be carried out from horses in Siberia (Russia) and China. To date, there is limited historic record that horses may have been transported to North America with Russian and Chinese explorers and merchants [46].

Prior to our study, only three complete mtDNA sequences were described from domestic and feral horses living in North America including one American Paint horse (haplogroup L), one Chincoteague pony (haplogroup A), and Saddlebred (haplogroup N) [23]. There are four additional almost complete mtDNA sequences obtained from Appaloosa, two Bashkir Curly horses, and American Paint horse [30]. However, the last sequences are problematic to use for the comparative study of the North American horses due to a 232-bp gap in their control regions.

In addition to the only entire mitochondrial genome sequences of a pony from Chinoteague Island our work provided three complete mtDNA sequences from the North American feral horses and national parks. Considering a very limited data on the mitochondrial genome diversity from feral equid populations in North America our study highlights the necessity for a survey of mitochondrial genome variability of feral horses living on federal lands to reveal what this data can tell us about their origins and history.

In terms of variability, the STR data shows similar results to what was found with the mtDNA data, that is, the TRNP herd has low genetic variability. Feral horse populations, on average, have levels of heterozygosity that are essentially the same as those of domestic horse breeds but the TRNP heterozygosity levels are well below those of both (Table 4). There are feral herds with lower heterozygosity values but in all cases, these are herds with very small population sizes or that have undergone severe recent bottlenecks [10, 11]. Very few domestic horse breeds have heterozygosity values as low as that of the TRNP herd and these also are populations with very low population size or with high levels of inbreeding [5]. Allelic diversity of feral herds is somewhat lower than that of domestic breeds, which is primarily a function of population size (few feral herds have herd sizes greater than 500 while most breed populations number in the thousands). Allelic diversity in the TRNP is well below the feral mean (and even further below the domestic breed mean) despite a relatively large sample size, which is associated with allelic diversity measures (Table 4). All variability measures are consistent with a recent population bottleneck which fits the known history of the herd. Unlike the mtDNA data where the observed sequences were unique or very rare, all STR alleles match known alleles from domestic horses.

The STR results shed little or no light on the origins of the TRNP herd. In the RML tree the TRNP clustered with an Iberian breed, the Garanno, which is a breed that has not contributed significantly to horse populations of North America [2]. The PCoA (Fig 3) analysis and the STRUCTURE analysis (Figs 4 and 5) show that the herd is clearly distinct from all domestic breeds with which it was compared. We also made some comparison with the other feral herds that were as geographically close to North Dakota as possible and none of these was remotely similar to the TRNP herd [47]. The possibility of a connection to a Russian breed such as the Yakutian horse that was suggested by the mtDNA data was not borne out by the nuclear DNA data. The Yakut horse was included in the PCoA analysis as was the Mongolian Horse and was very distinct from the TRNP herd (Fig 3; YAKT). The probable explanation for these results is that the TRNP herd is of mixed domestic breed origins with the subsequent loss of genetic variation due to a population bottleneck.

Our research has provided new information relevant to stewardship of the herd of feral horses at TRNP. Estimates of mitochondrial and nuclear genetic diversity lower than that observed for most feral and domestic breeds are cause for concern. To mitigate genetic diversity issues, it is recommended that new genetic stock be introduced to the park herd. A putative goal would be to elevate nuclear and mitochondrial genetic diversity of the TRNP herd to mean values observed among large (n<500 individuals) BLM herds or domestic breeds. Given the tepid results from prior attempts to introduce stallions to the herd [6], the park should consider introducing open mares that would be guaranteed to contribute to the next generation. Though the park’s horse herd is smaller than most BLM herds, a repeated introduction strategy could be implemented to allow gains in diversity to outpace genetic drift associated with small populations. In keeping with the park’s forage allocation model [48] a small (<90 individuals) genetically augmented herd could be maintained with higher genetic diversity than observed in the current closed herd. If the herd is to be maintained as a breeding population, then adaptive management, informed by ongoing genetic monitoring, should be conducted to ensure genetic integration at the herd and individual level.

Our efforts to identify specific herd origins through mtDNA analyses were unsuccessful. However, our determination that the herd is composed of at least two maternal sources, and three unique mitochondrial haplotypes, is informative and should be incorporated into management planning. Culling strategies should be modified to ensure that the existing maternal lineages are preserved and that any new maternal linages are maintained, following introductions.

The inconclusive findings resulting from RML, PCoA, and Bayesian clustering techniques reflect the herd’s history of bottlenecks followed by isolation and genetic drift. In essence, it appears that the herd’s nuclear genetic diversity has been reduced primarily to common alleles shared by many breeds and that allele frequencies have been modified by inbreeding to the extent that strong assignments cannot be supported by analysis of these few STR loci. Perhaps new technologies, such as high density single nucleotide polymorphism assays, could be employed in future analyses to reveal nuclear genomic relationships that resolve questions of ancestry, breed associations, recessive conditions, and other genetic predispositions of the herd. Given the unique mitochondrial lineages identified here, future comparative studies of horse mtDNA genome variation among feral horses and domestic breeds in North America may also help shed light on the origins of the TRNP herd. However, regardless of ancestry or conservation value of lineages, both of which remain unknown, immediate management efforts should focus on improving the genetic health of the herd.

## Supporting information

S1 Table Genetic variation in the mitochondrial genomes in three horses from Theodore Roosevelt Park carrying different mtDNA lineages. The sequences were aligned with the horse reference mtDNA sequence (GenBank accession number X79547).
(DOCX)

S2 Table SNPs in the GenBank mtDNA sequences closely related to the complete mtDNA sequences of the TRNP horses. (XLSX)
