# Supplementary material for: Genetic diversity and origin of the feral horses in Theodore Roosevelt National Park
Source: PLoS One. 2018 Aug 1;13(8):e0200795. doi: 10.1371/journal.pone.0200795 (PMC6070244; doi:10.1371/journal.pone.0200795)
Supplement: S1 Table — The sequences were aligned with the horse reference mtDNA sequence (GenBank accession number X79547). (DOCX) [file pone.0200795.s001.docx]

**S1 Table.** Genetic variation in the mitochondrial genomes in three horses from Theodore Roosevelt Park carrying different mtDNA lineages. The sequences were aligned with the horse reference mtDNA sequence (GenBank accession number X79547).

| **Gene** | **Start - stop positions** | **Horse 201016 (L2)** | **Horse 201346 (L1)** | **Horse 201230 (B)** |
| --- | --- | --- | --- | --- |
| tRNA-Phe | 1 - 70 |  |  |  |
| 12S rRNA | 71 - 1045 | 158A>G 356T>C 357.1C 960T>C | 158A>G 356T>C 357.1C 960T>C | 158A>G 356T>C 357.1C 858T>C |
| tRNA-Val | 1113 - 2693 | 1374A>G 1386delT 2227delT | 1374A>G 1386delT 2227delT | 2227delT |
| tRNA-Leu | 2694 - 2768 |  |  |  |
| ND1 | 2771 - 3727 | 2788C>T 2899T>C 3517C>T | 2788C>T 2899T>C 3517C>T |  |
| tRNA-Ile | 3727 - 3795 |  |  |  |
| tRNA-Gln | 3793 - 3865 |  |  |  |
| tRNA-Met | 3868 - 3936 |  |  |  |
| ND2 | 3937 - 4977 | 3942T>C 4062T>C 4536A>G 4646T>C 4669T>C | 3942T>C 4062T>C 4536A>G 4646T>C 4669T>C | 4062T>C |
| tRNA-Trp | 4976 - 5045 |  |  | 5016C>T |
| tRNA-Ala | 5121 - 5193 | 5098T>A | 5098T>A |  |
| tRNA-Asn | 5121 - 5193 |  |  |  |
| origin of L strand replication | 5194 - 5225 |  |  |  |
| tRNA-Cys | 5226 - 5293 | 5240delA 5279delA | 5240delA 5279delA |  |
| tRNA-Tyr | 5294 - 5360 |  |  |  |
| COX1 | 5362 - 6906 | 5529C>T 5817A>G 5886C>T 6006G>A 6309T>C 6786G>A | 5529 C>T 5817A>G 5886C>T 6006G>A 6309T>C 6786G>A | 5931T>C 6786G>A |
| tRNA-Ser | 6904 - 6972 |  |  |  |
| tRNA-Asp | 6981 - 7047 | 7003G>A | 7003G>A |  |
| COX2 | 7048 - 7731 | 7518A>G 7668A>G | 7518A>G 7668A>G | 7629T>C |
| tRNA-Lys | 7735 - 7802 |  |  |  |
| ATP8 | 7804 - 8007 | 7902T>C 8007G>A | 7902T>C 8007G>A |  |
| ATP6 | 7965 - 8645 | 8060C>T 8303A>G 8321C>T 8360G>A 8567C>T | 8060C>T 8303A>G 8321C>T 8360G>A 8567C>T |  |
| COX3 | 8645 - 9427 | 9241A>G | 9241A>G |  |
| tRNA-Gly | 9429 - 9497 |  |  |  |
| ND3 | 9498 - 9842 | 9686G>A | 9686G>A |  |
| tRNA-Arg | 9845 - 9913 |  |  |  |
| ND4L | 9915 - 10211 | 9953A>G 10112T>C 10125C>G 10126G>C | 9953A>G 10112T>C 10125C>G 10126G>C | 9963G>A |
| ND4 | 10205 - 11581 | 10216T>C 10294T>C 10378C>T 10423G>A 10615C>T 10828G>C 10829G>C 11242C>T 11545A>G | 10216T>C 10294T>C 10378C>T 10423G>A 10615C>T 10828G>C 10829G>C 11242C>T 11545A>G | 10737T>G 10766G>A 10828G>C 10829G>C 10834T>C 11242C>T |
| tRNA-His | 11583 - 11651 |  |  | 11617A>G |
| tRNA-Ser | 11652 - 11711 | 11684C>T 11695C>T | 11684C>T 11695C>T |  |
| tRNA-Leu | 11713 - 11782 |  |  |  |
| ND5 | 11789 - 13603 | 11844T>C 11881G>A 12121C>T 12202G>A 12769T>C 12898T>C 12952C>T 13051T>C 13335A>G 13522A>G | 11844T>C 11881G>A 12121C>T 12202G>A 12769T>C 12898T>C 12952C>T 13051T>C 13335A>G 13522A>G |  |
| ND6 | complement(13587 - 14114) | 13687G>A 13711G>A 13744G>T 13998T>G | 13687G>A 13711G>A 13744G>T 13998T>G | 13687G>A 13711G>A 13744G>T 13998T>G |
| tRNA-Glu | complement(14115 - 14183) | |  |  |
| CYTB | 14188 - 15327 | 14805T>C 14997A>G 15315T>C | 14805T>C 14997A>G 15315T>C | 14910T>C |
| tRNA-Thr | 15328 - 15401 |  |  |  |
| tRNA-Pro | 15403 - 15468 |  |  |  |
| control region | 15469 - 16660 | 15494T>C 15495T>C 15496A>G 15534C>T 15602C>T 15603T>C 15649A>G 15720G>A 15771C>T 15870C>T 15871C>T 15956A>G 15974C>T 16068T>C 16103C>T 16145G>A 16371T>C 16407delC 16629A>G | 15494T>C 15495T>C 15496A>G 15534C>T 15602C>T 15603T>C 15649A>G 15720G>A 15771C>T 15827A>G 15870C>T 15871C>T 15956A>G 15974C>T 16068T>C 16103C>T 16371T>C 16407del**C** 16629A>G | 15495T>C 15650A>G 15666G>A 15720G>A 15826A>G 16055A>G 16079A>G 16111G>A 16113G>A 16371T>C 16391C>T 16407delC |
